# Parents' information needs and their recommendations for effective sexuality education to children

**DOI:** 10.3389/fpubh.2025.1653924

**Published:** 2025-09-26

**Authors:** Neelam Punjani, Shannon D. Scott, Amber Hussain

**Affiliations:** University of Alberta, Edmonton, AB, Canada

**Keywords:** sexuality, sex, education, CSE, parent, caregiver, resources

## Abstract

**Background:**

As children undergo significant developmental transitions, comprehensive sexuality education (CSE) becomes essential. Despite global evidence supporting early CSE, many parents feel underprepared to address topics such as puberty, consent, gender identity, and digital safety with their children. This study explores parents' information needs, barriers, and recommendations for effective sexuality education tools.

**Methods:**

A community-based participatory research (CBPR) approach guided six focus group discussions (FGDs) with 30 diverse Canadian parents of children aged 0–18 years, conducted between January 2023 and March 2023. Discussions were audio-recorded, transcribed, and thematically analyzed using Braun and Clarke's inductive approach. Themes were validated through member checking and dual independent coding using NVivo.

**Results:**

Three major themes emerged from the data. First, parents expressed strong need for sexual health education support, including a desire for age-appropriate, inclusive, and culturally relevant resources, along with improved access to trustworthy information. Many felt underprepared to initiate conversations and called for clear guidance tailored to children's developmental stages. Second, parents prioritized a range of core content areas, such as puberty, hygiene, consent, healthy relationships, gender identity, and digital safety and highlighted the emotional and practical challenges of addressing these topics confidently. Third, parents recommended diverse, user-friendly formats for delivering sexual health education, including short digital videos, searchable websites, visual tools like charts and infographics, storybooks, multilingual materials, and structured workshops for both parents and children. Across all groups, there was a shared call for practical tools that are timely, culturally attuned, and capable of supporting value-aligned, ongoing conversations in the home.

**Conclusion:**

Canadian parents face different barriers to delivering effective sexuality education, shaped by cultural norms, informational gaps, and discomfort with sensitive topics. To empower parents as primary educators, there is a critical need for co-designed, accessible, and culturally inclusive resources that reflect diverse parenting contexts. Future efforts should explore how policy-level interventions particularly within education systems can support these collaborative efforts among families, schools, and healthcare providers to promote informed, age-appropriate, and inclusive sexual development.

## 1 Introduction

As children grow, they undergo significant physical, emotional, cognitive, and social changes that shape their understanding of themselves and their interactions with others. These developmental transitions mark a critical period for initiating conversations about sexuality, identity, consent, and bodily autonomy. According to the Sexuality Information and Education Council of the United States (1), comprehensive sexuality education (CSE) should begin as early as age five, encompassing topics such as personal safety, respect for personal space, consent, and health. These early discussions are essential in helping children make sense of the physical and emotional changes they experience (2, 3).

Sexuality education is increasingly recognized in global public health and policy frameworks as a fundamental human right (4). Evidence suggest that CSE contributes to reduce negative outcomes, including lower rates of teenage pregnancy, decreased sexually transmitted diseases (STDS), reduced school dropouts rates, and decreased substance use (5). Sexual health education also plays a vital role in child development by helping children explore their identities, gain knowledge, and build confidence and self-advocacy (6).

Despite this evidence, CSE delivery often remains limited to school-based models, overlooking the critical role of families in shaping children's early understanding of sexuality (7, 8). Across global contexts, these barriers include stigma, cultural and religious taboos, discomfort discussing sexual topics, and limited parental knowledge or confidence (9, 10). As a result, children and youth often turn to the internet, friends or pornography for sexual health information which may be inaccurate or harmful (11–14).

Parents play an essential, though often under-supported, role in their children's sexual health education. Unlike school-based programs that adopt a one-size-fits-all approach, parents have the opportunity to tailor discussions to their child's unique developmental stage and family values (6, 15). Research indicates that both children and parents express a willingness to engage in conversations about sexual health, particularly when trust and openness are present (16).

However, many parents feel ill-equipped due to lack of accessible resources, uncertainty about appropriate timing and language, and anxiety about doing harm (17–21). For parents of children with unique circumstances, the challenges are even more complicated due to lack of accessible resources (3, 22).

In Canada, the need for parent-supported sexuality education is particularly relevant given its multicultural population, decentralized education systems, and inconsistent sex education curricula across provinces (23). Although national frameworks such as the Canadian Guidelines for Sexual Health Education exist, implementation varies significantly across jurisdictions, and many parents report receiving little formal support or guidance in navigating these conversations (24). Furthermore, Canada's cultural diversity, including a significant immigrant population presents additional challenges for families, such as language barriers, culturally mismatched educational materials, and intergenerational differences in comfort and understanding of sexual health topics (25).

To address these challenges, a growing body of research has explored how tailored educational tools such as visual aids, mobile applications, and culturally relevant materials can support parents in delivering accurate and value-aligned sexual health education at home (26). Yet, limited studies have directly engaged Canadian parents to understand their specific informational needs, preferred resource formats, and cultural considerations (18, 27, 28).

The objective of this study is to explore Canadian parents' specific informational needs regarding sexual health education for their children. By directly engaging parents, the study contributes to the design tools that reflect the real-world diversity, values, and practical concerns of Canadian context. Our research builds upon and extends recent scholarship emphasizing inclusive and context-sensitive approaches to sexuality education (7, 8, 29–31).

## 2 Research methodology

### 2.1 Study design and objectives

This qualitative study employed a community-based participatory research (CBPR) design to explore parents' experiences and informational needs related to sexual health education. CBPR was selected to actively engage participants as co-creators of knowledge, ensuring that their voices and cultural contexts informed the data collection and interpretation process. The design facilitated a collaborative, iterative approach that prioritized cultural traditions, religious beliefs, and socioeconomic realities ensuring findings were grounded in the lived experiences of diverse families (32).

### 2.2 Study setting

The study was conducted virtually across six focus groups, using Zoom to allow participation from parents residing in Canada. Although online, the setting was intentionally designed to be inclusive, with sessions scheduled according to participants availability to accommodate parents' preference. Each session was moderated by trained qualitative researcher (NP) with expertise in sexual health. The use of Zoom was chosen for its accessibility, cost-effectiveness, and ability to reach a geographically diverse sample without the constraints of travel or childcare. However, we acknowledge limitations, including reduced capacity to observe nonverbal cues, occasional connectivity issues, and potential privacy concerns in shared home environments.

### 2.3 Participant recruitment and sampling

Participants were recruited through purposive and snowball sampling techniques to ensure demographic diversity and relevance to the study objectives. Recruitment strategies included digital flyers distributed via parenting groups, community organizations, school newsletters, and social media platforms. Interested individuals contacted the research team via email and were screened for eligibility. Six focus groups were conducted with five participants each, for a total sample of 30 parents. Recruitment continued until data saturation was achieved. All individuals who expressed interest participated in the study; there were no refusals or dropouts (A full demographic profile is presented in Section 3.1).

### 2.4 Study population and eligibility criteria

Participants were eligible for inclusion if they met the following criteria:

1. Were parents of at least one child aged 0–18 years.
2. Residing in Canada.
3. Could speak and read English.
4. Were willing to discuss their experiences about sexual health education.

All participants were required to provide informed written and verbal consent prior to participation. Individuals not currently parenting or not residing in Canada were excluded from the study.

### 2.5 Data collection process

We conducted six FGDs via Zoom, with groups consisting of five to six participants. Each session was audio-recorded and lasted approximately 60–90 min. Field notes were taken during and immediately after each session. A semi-structured discussion guide for FDG was developed and pilot-tested with two parents (refer to Appendix A). The guide directed discussions on (1) Parents' perspectives on effective sexuality education, (2) Challenges in discussing sexual health with children, (3) Preferred sources of information for sexual health education, (4) Recommended resources to facilitate meaningful conversations.

FGDs were conducted between January 2023 and March 2023. All focus groups were facilitated by the lead author (NP), a female PhD sexual and reproductive health researcher with qualitative research experience. No prior relationships existed between the researcher and participants before recruitment and data collection. No repeat FGDs were conducted with participants. Only participants and the researcher were present during each Zoom session; no non-participants were in attendance.

All discussions were audio-recorded, transcribed, and anonymized. Researchers maintained reflexive logs throughout to track observations and emerging themes. Inclusive facilitation techniques, such as validating diverse viewpoints and encouraging equal participation, minimized potential power imbalances in discussions. Participants had the option to skip questions or choose a private interview format, though none opted for one-on-one discussions. To address ethical concerns around digital data collection, Zoom sessions were password-protected and recordings were stored on encrypted drives accessible only to the research team. clarifications were sought during the sessions, and member-checking occurred in real time.

### 2.6 Data analysis

Data analysis followed an inductive thematic approach (33) six-phase framework. This approach is widely used in qualitative research to identify, analyze, and report patterns within data without imposing pre-existing theoretical frameworks. The six phases included: (1) familiarization with the data; (2) generating initial codes; (3) searching for themes; (4) reviewing themes; (5) defining and naming themes; and (6) producing the report.

Transcripts were cross-checked for accuracy, and open coding was applied to develop descriptive categories. Codes were then reviewed and refined into broader themes through constant comparison and researcher discussion. For instance, initial codes such as “*need for age-specific guidance*,” “*lack of inclusive resources*,” “*cultural mismatch*,” and “*difficulty accessing reliable materials*” were conceptually clustered and refined through collaborative analysis into the broader theme **“**Parents' Needs for Sexual Health Education Support.” Coding memos and shared documents tracked the evolution of themes. The final themes highlighted parents' recommendations for sexuality education and the types of resources they find valuable.

To enhance reliability, two independent researchers (NP and TL) coded the data separately, resolving discrepancies through discussion. NVivo 12 software facilitated data organization, while Microsoft Excel supported demographic analysis. Member checking with six participants confirmed the credibility of interpretations.

### 2.7 Positionality

The research team comprised individuals with professional backgrounds in sexual health, education, child health, and public health. Two of the lead researchers identified as parents themselves and brought lived experience to the project, while others identified as educators and advocates for equity-focused, culturally inclusive health programming. We acknowledge that our social locations such as gender, mixed-ethnicity, university-affiliated professionals may have influenced how we interpreted data and interacted with participants. To maintain reflexivity, the team engaged in regular debriefing sessions, kept individual and team reflexive journals during data collection and analysis to remain grounded in participants' perspectives.

### 2.8 Research rigor

To ensure the trustworthiness and rigor of this study, we adhered to Lincoln and Guba's (34) qualitative research criteria. Triangulation was implemented through peer debriefing, member checking, and reflective journaling, allowing for multiple perspectives to validate findings. Detailed descriptions of research settings and participant demographics were provided to enhance transferability, ensuring that the results could be applied to similar contexts. To support dependability, audit trails were maintained to document coding decisions, analytical processes, and thematic development, offering transparency and consistency in data analysis. Finally, systematic reflexivity was practiced throughout the research process, ensuring that findings accurately reflected participants' experiences rather than researcher biases, thereby reinforcing confirmability.

### 2.9 Ethical considerations

Ethics approval was secured from the University of Alberta Ethics Review Board (Pro00124683). Before participation, all parents provided written informed consent, with additional verbal consent recorded to confirm voluntary engagement and adherence to ethical guidelines. Participants were informed of the researcher's background and purpose of the research.

## 3 Results

### 3.1 Participant demographics

A total of 30 parents participated in the study, divided across six focus groups. The sample was diverse in terms of country of birth, years lived in Canada, age, gender, marital status, education, and household income (refer to Table 1 for consolidated summary of participant demographics).

**Table 1:** Demographic profile of participants (*n* = 30).

**Characteristics**	**Catogories**	***n***	**%**
**Country of birth**	Born in Canada	18	60
	Immigrated to Canada	12	40
**Years in Canada (for immigrants)**	Less than 5 years	10	33
	5–15 years	12	40
	More than 15 years	8	27
**Age group**	20–30 years	8	27
	31–40 years	10	33
	41–50 years	7	23
	51+ years	5	17
**Gender**	Female	22	73
	Male	6	20
	Non-binary	2	7
**Marital Status**	Married	24	80
	Common-law	4	13
	Divorced	2	7
**Education level**	High School	3	10
	College Certificate	10	33
	University Degree	17	57
**Household income**	Less than $40,000	6	20
	$40,000–$60,0000	10	33
	$60,0000–$80,000	8	27
	$80,000+	6	20

#### 3.1.1 Theme 1: Parents' needs for sexual health education support

This theme centers on the foundational needs of parents as they navigate when, how, and what to communicate to their children. A large number of parents expressed a strong desire for clear, age-appropriate, and inclusive resources to help guide these discussions. Many also reported difficulties in finding reliable and centralized sources of information they could trust. Additionally, they emphasized the importance of having materials that are culturally relevant and available in multiple languages to reflect the diversity of their families.

##### 3.1.1.1 Subtheme 1.1: foundational needs for age-appropriate and inclusive information

Across the focus groups, a strong consensus emerged around parents' desire for accessible, accurate, and culturally inclusive sexual health resources. These needs were not only content-based but also deeply tied to emotional readiness and trust. Parents emphasized challenges due to limited time, personal discomfort, and insufficient knowledge, which prevented them from effectively educating their children.

One participant voiced,

“*I don't feel equipped… like I don't have the right words to start these conversations, and then the time just passes*” (FGD2, P6).

Another parent added,

“*Even if I want to talk, I'm not sure how. I need a guide that says, ‘this is appropriate at 7, this at 10…'*” (FGD3, P3).

Others pointed to gaps in current resources, especially in addressing LGBTQ+ identities and culturally diverse experiences:

“*Most books don't talk about LGBTQ*+ *or they just ignore cultural backgrounds. I need something that reflects my family and still gives good info*” (FGD5, P2).

A mother noted frustration in finding materials that align with her values but also educate her child broadly:

“*How do I find a book for say an eight-year-old with trans children in it?*” (FGD1, P4).

There were repeated calls for multi-level, age-stratified materials. One participant said,

“*Can we just have a clear path? Like a chart… what to talk about at each stage?*” (FGD4, P1).

Another added,

“*I'd like resources that talk to my kid but also teach me how to talk to them*” (FGD6, P3).

##### 3.1.1.2 Subtheme 1.2: difficulty accessing reliable resources

Beyond content clarity, parents raised concerns about the lack of trustworthy and centralized resources. Many relied on improvised methods or internet searches due to a lack of clear alternatives:

“*I really don't have any kind of specific resources that I use... then I'll probably Google it.”* (FGD3, P2).

Concerns over misinformation were frequent. Parents wanted expert-backed material:

“*I need to take [in] information and approaches that have some basis in research*.” (FGD1, P4).

##### 3.1.1.3 Subtheme 1.3: cultural and linguistic accessibility

Some parents, especially from ethnocultural backgrounds, shared the need for cultural relevance and language accessibility in sexual health education. One participant said,

“*So it should be culture based*.” (FGD3, P3).

“*Well, I would hope that it would be in multiple languages*. Another participant added,

*English is not everyone's first language*.” (FGD3, P4).

Some parents also noted how misinformation and silence shaped intergenerational knowledge gaps:

“*Some mothers don't fully explain what a menstrual period is. Instead, they just hand over a cloth to use, saying it's sacred, without giving any real information*.” (FGD1, P2).

#### 3.1.2 Theme 2: priorities in sexual health content for children

This theme outlines what parents consider essential content areas in sexual education. Many parents emphasized the need for guidance on how to talk about bodily changes during puberty, including related hygiene habits. They also highlighted the importance of having tools to teach children about consent, personal boundaries, and healthy relationships. Some parents expressed a desire for support in having inclusive conversations about gender identity and sexual diversity. In addition, they wanted strategies to help navigate digital safety and address the influence of online content on their children.

##### 3.1.2.1 Subtheme 2.1: specific guidance on puberty, hygiene, and physical changes

Many participants emphasized on the need of guidance on when and how to discuss bodily changes, particularly during puberty. As one participant explained,

“*I use correct terms for body parts, but when should I start talking about periods or wet dreams?*” (FGD3, P4).

Another added,

“*I don't want to scare her, but she should know what will happen to her body before it happens*” (FGD1, P2).

Concerns included how to convey non-judgmental, respectful language:

“*I don't want her to feel ashamed about periods or boobs. Just factual*” (FGD4, P5).

Some parents also stressed the need of addressing hygiene habits alongside puberty:

“*There's no resource that shows the transition, like now they need deodorant, now shaving comes up. We wing it*” (FGD6, P1).

##### 3.1.2.2 Subtheme 2.2: consent, boundaries, and healthy relationships

Consent emerged as a particularly conflicted but urgent topic. One mother noted,

“*It's tricky… you tell them they have autonomy, but then you also say ‘eat your vegetables before dessert', it's confusing*” (FGD2, P3).

Parents wanted resources that not only educated children but also helped reconcile daily parenting with consent education.

Another parent emphasized,

“*Boundaries are bigger than sex. My child should know she can say no to a hug she doesn't want*” (FGD4, P6).

There was also interest in tools for addressing healthy relationships, as echoed by a participant:

“*It's not just body stuff. They need to know what respect looks like in a friendship or dating*” (FGD5, P2).

##### 3.1.2.3 Subtheme 2.3: gender, sexuality, and diversity

Parents expressed both curiosity and hesitation in addressing gender identity and sexual orientation. One participant said,

“*I'm learning too. I don't want to say the wrong thing. Maybe something with definitions and how to explain them to kids?*” (FGD6, P4).

Acceptance and inclusivity were frequently highlighted:

“*Kids come home with questions we didn't have to answer when we were kids. I want her to be kind, open, but I need the words*” (FGD1, P3).

##### 3.1.2.4 Subtheme 2.4: digital safety and online influence

Parents expressed considerable anxiety about their children's exposure to inappropriate content online. One parent shared,

“*He uses SnapChat. And I don't even know who he talks to. It freaks me out*” (FGD3, P2).

Others worried about body image distortion and misinformation online:

“*My daughter watches videos about diet stuff and body hacks… I can't tell if it's helping or harming her*” (FGD5, P4).

The tension between digital freedom and parental discomfort was a recurring conflict. While many parents acknowledged the inevitability of online exposure, they simultaneously felt unequipped to manage it resulting in restrictive or inconsistent approaches that may undermine trust. There was a strong call for tools to help navigate online risks,

“*A checklist would be good. Like, here's what to talk about before letting them get Instagram*” (FGD6, P1).

#### 3.1.3 Theme 3: recommendations for effective resource design and delivery

The last theme presents parent-suggested formats for delivering sexual health content. Many parents proposed using digital platforms and social media to reach both parents and children in engaging and accessible ways. They also recommended visual and print-based tools, such as storybooks, charts, and infographics, to help guide conversations at home. Media campaigns and public messaging were seen as useful for normalizing discussions about sexual health in everyday settings. Finally, some parents also suggested structured learning opportunities through schools or community workshops that could support both youth and caregivers in learning together.

##### 3.1.3.1 Subtheme 3.1: digital platforms for engaging learning

Many parents emphasized the practicality and widespread use of digital tools, especially social media as avenues to receive and share sexual health information. As one parent put it,

“*We are all on Facebook or Instagram… If there was a reliable page posting short tips, I'd follow it and share with others*” (FGD3, P2).

Participants noted the bite-sized, engaging nature of digital media as a strength:

“*Videos are easy, and my kids are on YouTube already. If something came up there, even I'd learn from it*” (FGD4, P4).

Several mentioned that short video clips (10–15 min) or animated explainers would work well for both parents and children.

Digital media was also praised for offering diverse perspectives and relatability. One parent said,

“*I follow some queer educators on Instagram… they explain gender and consent better than any book I've seen*” (FGD5, P6).

The ability to access such content in private or share it discreetly with children was seen as beneficial.

Parents emphasized a need for centralized, credible, and easy-to-use websites with searchable features. As one participant shared,

“*I want a site I can go to and type in: ‘how to talk about masturbation with my 11-year-old' and get answers right away*” (FGD2, P4).

Participants also suggested platforms include FAQs, visual tools, and even live chats or webinars with experts:

“*Sometimes I just want to ask a question and get an answer, like a doctor is there*” (FGD6, P1).

##### 3.1.3.2 Subtheme 3.2: visual and print-based resources for guided conversations

Parents widely supported the creation of infographics, pamphlets, and visual guides as supplemental tools. These formats were praised for being straightforward and easy to reference:

“*Sometimes I just want a chart or checklist on what to say at each age. Make it colourful, simple, and I'll use it*” (FGD2, P3).

Others agreed that visuals helped retain information:

“*Pamphlets with drawings stick in my head more than text. If I can look at a picture and remember the point, that's useful*” (FGD6, P5).

Several parents also emphasized the need for materials to be written in accessible language,

“*like at a grade 10 reading level, so it's not too academic or complicated, just something we can actually use at home*” (FGD1, P2).

There were repeated calls for multilingual resources:

“*I'd like something in my first language. Even basic translations so I can give it to my mom or aunt to help with my daughter*” (FGD4, P1).

Visual materials were seen as ideal for quick skimming, returning to topics, and for use during clinic visits or school meetings.

For parents with young children, storybooks were repeatedly cited as a trusted and effective method to introduce complex topics. As one participant said,

“*Kids love stories. If the main character is learning about body parts or boundaries, they learn without pressure*” (FGD3, P1).

Another parent shared,

“*We read together before bed. A book that casually teaches about puberty would be perfect*” (FGD6, P4).

The blend of relatable characters, simple narratives, and gentle visuals made storybooks a top choice for younger audiences. Graphic novels were also suggested as suitable for pre-teens and adolescents:

“*Comics or graphic books might get my son's attention more than a lecture*” (FGD2, P5).

##### 3.1.3.3 Subtheme 3.3: media and public campaigns to normalize conversations

Several parents mentioned that media moments, such as commercials, TV shows, or news items, often served as organic conversation starters with their children. One parent shared,

“*We were watching something where someone came out as gay. It gave me a reason to talk to my son about sexuality*” (FGD4, P3).

Others saw the potential for PSAs or informational ads to reinforce key messages:

“*I see ads for dental health. Why not sexual health? A quick reminder could really help*” (FGD1, P6).

Some parents felt that general media could normalize the conversation and reach both youth and adults at once.

##### 3.1.3.4 Subtheme 3.4: schools and structured learning for parents and youth

Many participants preferred structured and consistent learning environments, especially when their own comfort or knowledge levels were low. As one parent explained,

“*If my daughter gets a workshop at school, then I can ask her what she learned. It makes the conversation easier*” (FGD3, P4).

Some parents also expressed interest in parent-specific workshops to prepare them for these conversations:

“*I'd attend a short online class on how to talk about periods or dating. I need someone to walk me through it*” (FGD5, P1).

Several called for these programs to be culturally sensitive,

“*We need facilitators who understand our background*” (FGD6, P2),

and offered in multiple formats including in-person, online, or video-based.

The idea of creating community-based learning groups also came up:

“*It'd be nice to have a space with other parents, so we can share ideas and ask questions*” (FGD4, P5).

## 4 Discussion

This study demonstrates that parents have specific and multi-facet needs when it comes to teaching their children about sexual health. Parents in this study articulated a strong need for sexual health resources that are not only accurate, but also age-appropriate and easy to implement in everyday life. Rather than waiting for formal education settings, many parents expressed wanting a “roadmap” to know what's appropriate to discuss at different stages. This extends previous findings that focused on adolescent communication by offering insight into early childhood and middle-years communication needs (8). The desire for structured timing guidance echoes research showing that gay, bisexual, and queer adolescents desired earlier and clearer communication from caregivers but found that conversations often came too late or lacked inclusive language (8).

These findings align with Bronfenbrenner's Ecological Systems Theory (35), which highlights the influential role of parents within children's microsystems. Additionally, Social Cognitive Theory (36) supports the value of modeling and guided learning, highlighting the importance of parent-child conversations, visual tools, and relatable narratives as mechanisms to shape beliefs and behavior. Together, these frameworks suggest that educational tools must engage not only in cognitive comprehension but also family dynamics, parental confidence, and cultural context.

The importance of culturally relevant and linguistically accessible material was another recurrent theme. Several parents, especially those from immigrant or multilingual households, expressed frustration with existing resources that did not reflect their lived experiences. This reflects research which found that many sexual health materials are not translated and are often written at a reading level too advanced for some families, creating a gap in accessibility (31). However, our findings go beyond surface-level barriers, revealing a deeper need for emotional trust and cultural resonance, elements that are often missing in existing interventions. The inclusion of culturally grounded examples and language not only enhances comprehension but also increases emotional trust and relevance, key components emphasized in both ecological and cognitive-behavioral learning models.

One of the more delicate areas parents navigated was the topic of consent and bodily boundaries. While many parents valued teaching their children bodily autonomy, they struggled to do so without contradicting everyday practices such as enforcing hugs, bedtime routines, or personal hygiene expectations. These actions, although well-intentioned, often sent mixed messages about agency and control. This created a palpable tension between the desire to empower children with autonomy and the perceived need to maintain order, safety, and social norms. For some parents, this resulted in hesitation or avoidance preferring not to raise the issue of consent at all out of fear they might “get it wrong.” While literature suggests that tools like structured scripts and visuals can help reinforce self-advocacy, particularly for neurodiverse children (29), our study expands on this by highlighting the emotional labor and role conflict parents experience when trying to reconcile empowerment with authority.

Another critical area of need was in addressing gender identity and sexual diversity. While many parents showed willingness to support inclusive learning, they also reported not having the language or understanding to do so confidently. This aligns with the work of Martino et al. (7), who explored the challenges faced by parents and educators when trying to provide affirming and accessible sex education for youth with developmental disabilities and shows that the absence of LGBTQ+ content made families feel unprepared. To bridge this gap, inclusive education must extend beyond simply providing definitions, it must include narratives, scripts, and frameworks that reflect cultural sensitivity, developmental readiness, and the lived realities of diverse families. Without this, parents may continue to avoid these conversations out of fear, even when they support inclusion in principle.

Parents prioritized early, concrete education on puberty, hygiene, and bodily changes—particularly for children with developmental disabilities—often prompted by safety concerns or exposure. A recent study similarly found that autistic adolescents often received little or no education about bodily changes, and that visual aids, simplified language, and routine-based instruction were recommended to better support these learners (37). Yet, our findings suggest that having tools is not enough, parental emotional readiness and relational dynamics critically shape how and when those tools are used.

A key digital need identified by parents was the availability of reliable and user-friendly online tools. Many parents desired websites or platforms that could answer specific questions in real time, while maintaining accuracy and age relevance. This mirrors findings from a review that evaluated LGBTQI cancer patient resources online, which found most platforms to be disorganized, not inclusive, and hard to navigate prompting a call for centralized, evidence-based online hubs (30). Thus, building effective online tools requires not only good design but also embedding digital literacy and cultural navigation support.

Alongside usability, digital safety emerged as a growing concern. Parents sought practical support for preparing children to navigate online risks and manage interactions on apps like Snapchat and Instagram. This aligns with a legal and clinical review that called for structured digital safety tools such as checklists, media contracts, and content guidelines especially for families with neurodiverse children (38). Together, these insights reveal a widening gap between children's digital fluency and parents' preparedness. Our study suggests that this digital divide is not only technical, but emotional, rooted in fear, confusion, and a lack of confidence—requiring interventions that address both content and caregiver readiness.

Format preferences emerged clearly in the data. Many parents shared a preference for visual materials and print-based tools to facilitate conversations. Infographics, storybooks, and graphic novels were recommended as accessible, engaging formats that reduced discomfort and increased message retention. This preference is consistent with a study that developed a social-sexual curriculum for adults with neurodevelopmental disabilities, finding that visual resources significantly improved understanding and engagement (39). However, the effectiveness of these formats also lies in their ability to normalize sensitive topics. For instance, storybooks featuring relatable characters can help younger children internalize key messages without perceiving them as taboo. When well-designed, these formats serve a dual function: they inform the child while empowering the parent. In this way, visual resources bridge the cognitive and emotional gaps that often prevent families from addressing sexual health proactively.

While content and delivery were central, the how and where of receiving resources also mattered. Parents valued distribution from familiar community figures such as pediatricians, school counselors, or local facilitators. They expressed a strong interest in workshops or webinars designed specifically for caregivers, especially if offered in multiple languages or culturally tailored formats. This approach is supported by research that evaluated the role of community workshops in improving sexual health literacy and emphasized the success of culturally responsive, peer-led programming (40). By embedding content in trusted community channels, our study suggests that uptake is not only more likely, but also more sustainable because trust and social connection amplify engagement.

Finally, parents consistently stressed the need for timing guidance. Many had begun early conversations due to situational exposure. This reinforces previous findings that show early, proactive dialogue fosters trust and helps prevent adolescents from turning to unreliable online or peer sources (8). To support this, it is essential to provide age-appropriate, emotionally attuned resources that help caregivers align timing with both cognitive development and real-world exposure. When implemented effectively, this approach not only normalizes open dialogue but also empowers parents to engage with confidence and consistency.

### 4.1 Implications and recommendations

Our study confirms an urgent need for centrally curated, digital-first, culturally nuanced sexual health resources. However, these tools must go beyond simple information provision. They should include interactive guides, scripts, model conversations, checklists, and visual aids. This should include consideration for culturally appropriate adaptations that reflect gender norms and sensitivities in diverse Canadian communities. Tailoring messages for both boys and girls, in ways that resonate with cultural values, could significantly enhance uptake and usability.

Future research should explore which formats most effectively support parent-child communication, especially across diverse populations. Moreover, program evaluations should assess not only content accuracy, but also emotional resonance, usability, and adaptability in real-world family contexts.

### 4.2 Limitations

The study has a few limitations that should be acknowledged. Our study was conducted through FGDs, which may have influenced how parents responded. Some parents might have felt hesitant to voice differing or less common opinions, making it harder for those with minority viewpoints to elaborate on their thoughts. Group dynamics may have encouraged consensus or led participants to emphasize socially acceptable views, particularly around sensitive topics like gender identity or consent.

Additionally, most of the participants were parents of elementary-aged children, meaning their concerns and experiences may not fully capture those of parents raising teenagers. Expanding research to include parents of adolescents would provide valuable insights into their unique challenges.

While our study included a diverse range of participants, many discussions were framed by heteronormative experiences. This highlights the need to further explore how parents navigate sexual health education for children who are gender or sexually diverse, as their needs and barriers may differ. It is also important to consider how intersections of race, class, gender, immigration status and language fluency may have shaped participants' comfort levels in discussing certain topics during the sessions. Lastly, participants were recruited through community organizations and online platforms, which may have led to sampling bias potentially over-representing parents who are already more engaged or have stronger opinions about sexual health education.

## 5 Conclusion

This study highlights the multifaceted needs parents face in navigating sexual health education with their children. Parents consistently expressed a desire for clear, credible, and developmentally appropriate resources that reflect diverse identities and cultural contexts. They prioritized support in discussing puberty, hygiene, consent, relationships, gender identity, and digital safety, often feeling unprepared or unsupported in these areas.

To move from understanding to action, future initiatives should pilot co-designed digital tools that offer timely, evidence-based, and culturally relevant guidance. Schools and public health institutions should implement community-based, multilingual workshops that empower parents as trusted educators. Policymakers are encouraged to integrate parent voices into curriculum development and health promotion strategies, ensuring resources are accessible, inclusive, and adaptable to everyday parenting. By aligning resources with real-life family contexts through visuals, storytelling, expert-led platforms, and participatory formats interventions can bridge current gaps in confidence, knowledge, and trust. Ultimately, empowering parents as early sexuality educators strengthens both family communication and child wellbeing, making it a critical public health priority.

## Data Availability

The raw data supporting the conclusions of this article will be made available by the authors, without undue reservation.

## Appendix

### Focus group discussion guide

1. What is your understanding about comprehensive sexuality education? What does it entail and what are some components?
2. How comfortable would you feel talking about sexual health issues with your children and why?
3. What are your experiences about parent-child communication about sexuality education?
4. How often do you talk with your children about sexuality and sexual health?
5. How soon do you think one should start talking about sexual health with children? In your experience when did you first start talking with your kids?
6. What are some of the important topics that you think are considered part of comprehensive sexuality education?
7. What are some of the challenges or barriers that you face while talking about sexual health with your kids? And why?
8. What are some of the facilitators you think that helped you or will help you in talking about sexual health with your children?
9. What would make it easier to talk with your children about these topics?
10. What material and resources have you used to talk about sexuality?
11. What was your experience using these materials/resources?
12. What are some of the recommendations you can provide in developing digital translation resources for parents to encourage parents to talk about sexuality?
